# Cesarean section rates according to the Robson Classification and its association with adequacy levels of prenatal care: a cross-sectional hospital-based study in Brazil

**DOI:** 10.1186/s12884-023-05768-2

**Published:** 2023-06-20

**Authors:** Veridiana Monteiro Ramos Piva, Verena Voget, Luciana Bertoldi Nucci

**Affiliations:** 1 Health Sciences Post Graduate Program, Faculty of Medicine, School of Life Sciences, Pontifical Catholic University of Campinas, Av. John Boyd Dunlop, S/N - Jd. Ipaussurama, Campinas - São Paulo, CEP: 13060-904 Brazil; 2 Faculty of Medicine, School of Life Sciences, Pontifical Catholic University of Campinas, Av. John Boyd Dunlop, S/N - Jd. Ipaussurama, Campinas - São Paulo, CEP: 13060-904 Brazil

**Keywords:** Cesarean sections, APNCU index, Robson classification, Brazil, Mode of delivery, Epidemiology, Prenatal

## Abstract

**Background:**

The rate of Cesarean section (CS) deliveries has been increasing worldwide for decades. Brazil exhibits high rates of patient-requested CS deliveries. Prenatal care is essential for reducing and preventing maternal and child morbidity and mortality, ensuring women's health and well-being. The aim of this study was to verify the association between the level of prenatal care, as measured by the Kotelchuck (APNCU – Adequacy of the prenatal care utilization) index and CS rates.

**Methods:**

We conducted a cross-sectional study based on data from routine hospital digital records and federal public health system databases (2014–2017). We performed descriptive analyses, prepared Robson Classification Report tables, and estimated the CS rate for the relevant Robson groups across distinct levels of prenatal care. Our analysis also considered the payment source for each childbirth – either public healthcare or private health insurers – and maternal sociodemographic data.

**Results:**

CS rate by level of access to prenatal care was 80.0% for no care, 45.2% for inadequate, 44.2% for intermediate, 43.0% for adequate, and 50.5% for the adequate plus category. No statistically significant associations were found between the adequacy of prenatal care and the rate of cesarean sections in any of the most relevant Robson groups, across both public (*n* = 7,359) and private healthcare (*n* = 1,551) deliveries.

**Conclusion:**

Access to prenatal care, according to the trimester in which prenatal care was initiated and the number of prenatal visits, was not associated with the cesarean section rate, suggesting that factors that assess the quality of prenatal care, not simply adequacy of access, should be investigated.

## Background

Cesarean sections (CS) are surgical interventions usually recommended when a vaginal delivery would incur a higher risk of death for women and newborns. Examples of complications that increase such risk are antepartum hemorrhage, fetal distress, abnormal fetal presentation, and hypertensive disease [1]. It is increasingly more common that pregnant women, who are not affected by such complications, request delivery by CS for other reasons. Several studies show that this option is a global trend, being more pronounced in some countries, including Brazil [1–4].

A national hospital-based study with 23,894 pregnant women in Brazil showed an overall CS rate of 51.9% in 2011–12. When stratified according to health system, CS rates were 42.9% in national public healthcare (from a sample of 19,129 parturients) and 87.9% within private healthcare (4,765) [5]. There is no evidence that high rates of non-required CS deliveries, as seen in Brazil, are associated with lower mortality or morbidity for women or infants [6]. CS can also increase the risk of abnormal placentation and uterine rupture in future pregnancies, besides surgical adhesions, painful menses, endometriosis, and infertility [3].

Due to concerns by the medical community on the increasing CS rates, there are ongoing international efforts, supported by the World Health Organization (WHO) [7], to monitor and potentially reduce CS rates. The internationally accepted system to monitor and compare CS rates across delivery units is the Robson Ten Group Classification System [8, 9]. It categorizes every childbirth into one, and one only, group. The groups are mutually exclusive, inclusive, and clinically relevant. In recent studies, two of the original ten groups (Groups 2 and 4) have been split into four (2a and 2b, 4a and 4b) to allow for more granular analyses [10–12]. The parameters used to define each group are parity, previous CS, number of fetuses, fetal presentation, gestational age, and onset of labor (Table 1).

**Table 1:** Criteria for Robson groups (adapted from Robson, 2001 [13])

Group	Parity	Previous CS	Number of fetuses	Fetal presentation	Gestational age (weeks)	On set of labor
1	Nulliparous	N/A	Single	Cephalic	≥ 37	Spontaneous
2a						Induced
2b						Pre-labor CS
3	Multiparous	No				Spontaneous
4a						Induced
4b						Pre-labor CS
5		≥ 1				^a^
6	Nulliparous	N/A		Breech	^a^	
7	Multiparous	^a^				
8	^a^		Multiple			
9			Single	Transverse or oblique		
10				Cephalic	< 37	

*N/A* Not applicable

^*^indicates "all possibilities", "anything"

There is no “ideal” CS rate for each Robson group. Numbers depend on epidemiological factors, as well as the organizational and cultural context of each delivery unit [9]. Nevertheless, given the growing adoption of the Robson classification [8], it is increasingly possible to contrast and compare experiences between obstetric units [14]. In fact, to help the implementation of Robson classification all over the world, WHO provides a manual with guidelines and some typical ranges of CS rates [15].

Prenatal care could be an important factor influencing CS rates, as prenatal visits are not only essential for monitoring maternal and fetal health, but they also provide an opportunity for discussing and planning the delivery itself [16]. Given the substantial number of dimensions involved in prenatal visits that are not collected in data records, it is challenging to assess prenatal care systematically. The Adequacy of Prenatal Care Utilization (APNCU) index provides relatively simple criteria for assigning the prenatal care into four “adequacy” levels: inadequate, intermediate, adequate and adequate plus. The levels are based on the number of visits and gestational age at the first prenatal visit [17].

Thus, the aim of this study was to evaluate a potential association between the adequacy of prenatal care, as categorized by the APNCU index, and the rate of Cesarean deliveries across Robson groups in our institution. If there were an association between adequacy levels of prenatal care and lower CS rates, it could be a strategy worth considering in the ongoing initiatives related to reducing CS deliveries.

## Methods

### Study design, setting, and participants

This was a cross-sectional hospital-based study conducted at the PUC Hospital-Campinas in Campinas—São Paulo, Brazil – a tertiary care facility and teaching hospital that serves both public healthcare and privately insured patients. The study population included all records of women who gave birth at the aforementioned hospital from January 2014 to December 2017.

### Data source and variables

**Fig. 1:**
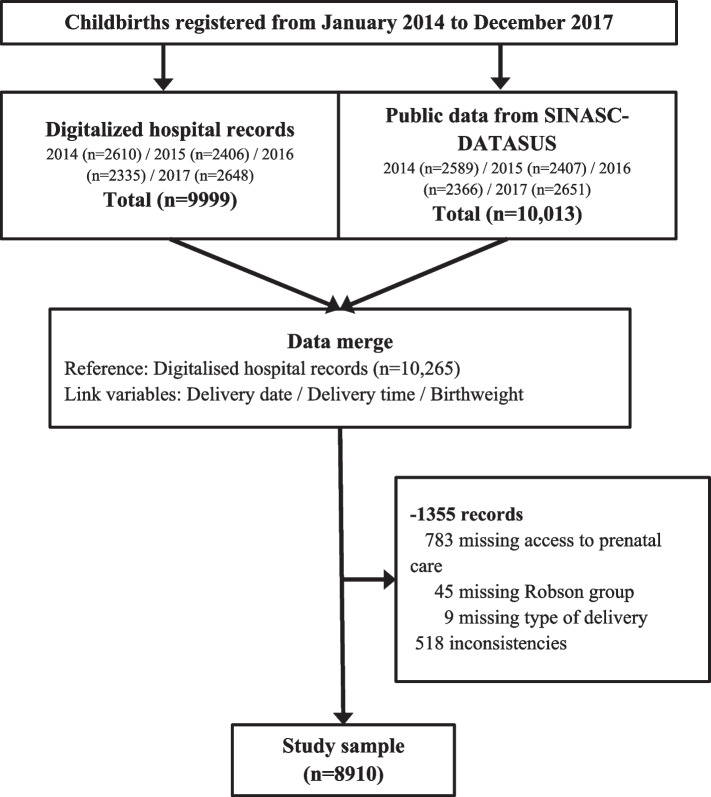
Flowchart to define the study sample. PUC Hospital—Campinas, 2014–2017

Data was extracted from routine hospital digital records and supplementary information was obtained from DATASUS/SINASC [18] (a national, publicly available health information system) and, where necessary, patient medical notes. Records of 837 births were excluded due to missing data, and 518 were excluded due to data inconsistencies between delivery date, delivery time and/or birth weight that could not be solved after further investigation with the hospital medical records. Figure 1 exhibits a flowchart with details on how the 8,910-record database was compiled.

The variables considered in this study were: method of delivery, number of prenatal visits and gestational age at first prenatal visit (used for assigning a APNCU category), payment source for each birth (either public healthcare or private insurers), year of delivery, maternal sociodemographic information (age, level of schooling, marital status, and ethnicity/skin color), and Robson group (which considers parity, previous CS, number of fetuses, fetal presentation, gestational age at labor, and onset of labor).

### Data processing and analysis

The CS rate for each variable was calculated, and Chi-square tests were performed to verify the association between these variables and type of delivery. Women were then categorized into one of the ten Robson groups and standard Robson Classification Report tables were prepared. Level of access to prenatal care was computed according to APNCU index based on Brazilian Ministry of Health categories: 1-Inadequate, for pregnant women who began antenatal care after the first trimester of pregnancy and/or attended less than three consultations; 2-Intermediate, pregnant women who started antenatal care during the first trimester and who had three to five consultations; 3-Adequate, pregnant women who commenced antenatal care during the first trimester, with six consultations; or 4-Adequate Plus, pregnant women who began antenatal care during the first trimester, with at least seven consultations [19].

To investigate association between CS rates and level of access to prenatal care by relevant Robson group and health system (payment source), adjusted CS rates and their 95% confidence intervals were calculated through logistic model. Robson Groups 1 to 5 and 10 were deemed the relevant ones for this analysis. Groups 1 and 2 encompass nulliparous women with single cephalic pregnancy at term. Groups 3 and 4 comprise multipara with single cephalic pregnancy at term and no previous Cesarean delivery. Group 5 consists of all multiparous women with single cephalic pregnancy at term and at least one previous uterine scar (CS). Group 10 is composed of all women with single cephalic pregnancy before term, including those with previous CS. Robson Groups 6, 7, 8, and 9 were not considered in this analysis because a CS delivery is usually the obstetric recommendation in such cases.

Analyses were performed using the statistical software SAS on Demand for Academics (SAS Studio version 3.8). The level of significance (α) adopted was 0.05.

## Results

Over the four-year period, a total of 8,910 births were evaluated: 2251 in 2014, 2177 in 2015, 2117 in 2016, and 2,365 in 2017. The mean (standard deviation) maternal age at delivery for the whole sample was 25.9 (6.6) years, with a minimum age of 11 years old and a maximum age of 51 years old. There were 7359 (82.6%) deliveries within the public health system (SUS) and 1551 (17.4%) were paid for by private health insurance.

The overall CS rate was 48.8%, with large differences between SUS and private health insurance (42,0% and 80,7%, respectively). Higher CS rates were observed among older (≥ 35 years) women (61.6%), with clear increase in this rate as age increases; with a higher level of schooling (71.7%); separated, divorced, or widowed (63.3%); classified as white (52.5%); and within private healthcare (80.7%). No statistically significant differences in CS rates were found between the different years of delivery, ethnicity/skin color for SUS and access to prenatal care for private health insurance (Table 2).

**Table 2:** Type of delivery total and by healthcare system according to year, maternal characteristics, healthcare system, and access to prenatal care

	**Healthcare System**								
	SUS			Private			**Total**		
	**Type of Delivery**			**Type of Delivery**			**Type of Delivery**		
**Characteristic**	**Normal n (%)**	**Cesarean n (%)**	***p*-value**	**Normal n (%)**	**Cesarean n (%)**	***p*-value**	**Normal n (%)**	**Cesarean n (%)**	***p*-value**
**Total**	4265 (58.0)	3094 (42.0)		299 (19.3)	1252 (80.7)		4564 (51.2)	4346 (48.8)	
**Year**									
2014	1056 (56.7)	805 (43.4)	0.100^a^	74 (19.0)	316 (81.0)	0.922^a^	1130 (50.2)	1121 (49.8)	0.153^a^
2015	1080 (59.6)	731 (40.4)		73 (20.0)	293 (80.0)		1153 (53.0)	1024 (47.0)	
2016	983 (56.3)	764 (43.7)		74 (20.0)	296 (80.0)		1057 (49.9)	1060 (50.1)	
2017	1146 (59.1)	794 (40.9)		78 (18.3)	347 (81.7)		1224 (51.8)	1141 (48.2)	
**Age (years)**									
11 – 14	57 (74.0)	20 (26.0)	< 0.001^a^	0 (0.0)	2 (100.0)	0.005^b^	57 (72.1)	22 (27.9)	< 0.001^a^
15 – 19	995 (66.4)	504 (33.6)		26 (33.3)	52 (66.7)		1021 (64.7)	556 (35.3)	
20 – 34	2846 (56.8)	2167 (43.2)		225 (19.4)	936 (80.6)		3071 (49.7)	3103 (50.3)	
≥ 35	367 (47.7)	403 (52.3)		48 (15.5)	262 (84.5)		415 (38.4)	665 (61.6)	
**Schooling**									
≤ Elementary I	115 (61.2)	73 (38.8)	< 0.001^a^	2 (20.0)	8 (80.0)	0.001^a^	117 (59.1)	81 (40.9)	< 0.001^a^
Elementary II	1231 (61.6)	766 (38.4)		16 (24.2)	50 (75.8)		1247 (60.5)	816 (39.5)	
High School	2712 (56.9)	2053 (43.1)		193 (22.4)	668 (77.6)		2905 (51.6)	2721 (48.4)	
Higher education	196 (50.1)	195 (49.9)		88 (14.3)	526 (85.7)		284 (28.3)	721 (71.7)	
**Marital Status**									
Separated, Divorced, or Widowed	29 (36.7)	50 (63.3)	< 0.001^a^	4 (25.0)	12 (75.0)	0.008^a^	33 (34.7)	62 (65.3)	< 0.001^a^
Single	1665 (62.2)	1010 (37.8)		57 (26.9)	155 (73.1)		1722 (59.6)	1165 (40.4)	
Married or Stable union	2550 (55.9)	2012 (44.1)		237 (18.0)	1081 (82.0)		2787 (47.4)	3093 (52.6)	
**Ethnicity/skin color**									
White	1663 (57.4)	1233 (42.6)	0.320^a^	156 (16.7)	778 (83.3)	0.005^b^	1819 (47.5)	2011 (52.5)	< 0.001^a^
Black	439 (54.9)	360 (45.1)		28 (25.4)	82 (74.6)		467 (51.4)	442 (48.6)	
Asian	15 (39.5)	23 (60.5)		1 (14.3)	6 (85.7)		24 (53.3)	21 (46.7)	
Brown	1893 (58.9)	1319 (41.1)		102 (24.3)	318 (75.7)		1995 (54.9)	1637 (45.1)	
Indigenous	6 (54.5)	5 (45.5)		0 (0.0)	1 (100.0)		6 (50.0)	6 (50.0)	
**Access to prenatal care**									
None	2 (22.2)	7 (77.8)	0.014^a^	0 (0.0)	1 (100.0)	0.228^a^	2 (20.0)	8 (80.0)	< 0.001^a^
Inadequate	937 (60.2)	619 (39.8)		57 (22.0)	202 (78.0)		994 (54.8)	821 (45.2)	
Intermediate	244 (60.3)	161 (39.7)		15 (25.4)	44 (74.6)		259 (55.8)	205 (44.2)	
Adequate	242 (60.8)	156 (39.2)		13 (26.5)	36 (73.5)		255 (57.1)	192 (42.9)	
Adequate Plus	2840 (56.9)	2151 (43.1)		214 (18.1)	969 (81.9)		3054 (49.5)	3120 (50.5)	

*SUS* National Health System (Acronym for Sistema Único de Saúde in Portuguese)

^a^Chi-square test

^b^Fisher Exact test

CS rate and number of deliveries by level of access to prenatal care were 80.0% (8 out of 10 women) for none, 45.2% (821 out of 1823) for inadequate, 44.2% (205 out of 464) for intermediate, 43.0% (192 out of 447) for adequate, and 50.5% (3120 out of 6174) for adequate plus category (Table 2).

Table 3 shows data of Robson groups, but because of the disparities in CS rates between private and public healthcare, data is split by healthcare system. Due to missing data, there were 43 deliveries within private healthcare and 12 within public healthcare whose Robson groups could not be determined.

**Table 3:** The Robson Classification Report Table, as recommended by the World Health Organization

Column												
**1**	**2**		**3**		**4**		**5**		**6**		**7**	
**Group**^**a**^	**Number of CS in Group**		**Number of women in group**		**Group Size**^**b**^** (%)**		**Group CS rate**^**c**^ **(%)**		**Absolute group contribution to overall CS rate**^**d**^** (%)**		**Relative contribution of group to overall CS rate**^**e**^** (%)**	
	**Public**	**Private**	**Public**	**Private**	**Public**	**Private**	**Public**	**Private**	**Public**	**Private**	**Public**	**Private**
1	236	160	972	234	13.2	15.2	24.3	68.4	2.6	1.8	7.6	12.9
2	626	329	1271	365	17.3	23.6	49.3	90.1	7.0	3.7	20.3	26.4
2a	337	38	982	74	13.3	4.8	34.3	51.4	3.8	0.4	10.9	3.1
2b	289	291	289	291	3.9	18.8	100.0	100.0	3.2	3.3	9.3	23.4
3	126	47	1347	140	18.3	9.1	9.4	33.6	1.4	0.5	4.1	3.8
4	278	96	826	130	11.2	8.4	33.7	73.8	3.1	1.1	9.0	7.7
4a	140	10	688	44	9.4	2.8	20.3	22.7	1.6	0.1	4.5	0.8
4b	138	86	138	86	1.9	5.6	100.0	100.0	1.5	1.0	4.5	6.9
5	1114	369	1653	387	22.5	25.1	67.4	95.3	12.5	4.1	36.0	29.6
6	69	34	72	34	1.0	2.2	95.8	100.0	0.8	0.4	2.2	2.7
7	106	40	113	40	1.5	2.6	93.8	100.0	1.2	0.4	3.4	3.2
8	103	50	123	52	1.7	3.4	83.7	96.2	1.2	0.6	3.3	4.0
9	14	2	14	2	0.2	0.1	100.0	100.0	0.2	0.0	0.5	0.2
10	419	118	965	160	13.1	10.4	43.4	73.8	4.7	1.3	13.5	9.5
Total	3091	1245	7356	1544	100.0	100.0	42.0	80.6	34.7	14.0	100.0	100.0
X	36	12	43	12	0.6	0.8	-	-	-	-	-	-

Group X: Unable to classify

*CS* Cesarean section

^a^ Groups descriptions are presented in the Methods section

^b^Group size (%) = number of women in the group / total number of parturient × 100

^c^Group CS rate (%) = number of CS in the group / total number of parturient in the group × 100

^d^Absolute contribution (%) = number of CS in the group / total number of parturient × 100

^e^Relative contribution (%) = number of CS in the group / total number of CS × 100

Within private healthcare, women in Group 5 were the largest group, accounting for 25.1% of all deliveries. This was closely followed by Group 2 (nulliparous women with single cephalic pregnancy at term who either had an induction of labor or a CS before the onset of labor) at 23.6%. Group 1 (nulliparous women with single cephalic pregnancy at term in spontaneous labor) was the third largest group accounting for 15.2% of deliveries. The largest relative contributors to the overall CS rate were the same groups in the same order, Group 5 (29.6%), Group 2 (26.4%) and Group 1 (12.9%). These three groups contributed for nearly 70% of cesarean deliveries within private care. Women in Groups 2 and 4 were further divided into the ones who had either (a) induced labor or (b) pre-labor cesarean deliveries. For privately insured parturients, in Group 2, 74 (20.3%) had induced deliveries (2a) and 291 (79.7%) pre-labor CS (2b). Group 4 had 44 (33.8%) inductions (4a) and 86 (66.2%) pre-labor CS (4b). Group 10 (single cephalic preterm deliveries) were 10.4% of all deliveries and contributed with 9.5% of cesarean deliveries (Table 3).

For the public healthcare system, women in Group 5 were also the largest group, accounting for 22.5% of deliveries. Followed by Group 3 (multiparous women with single cephalic pregnancy at term in spontaneous labor without previous CS) and Group 2, which accounted for 18.3% and 17.3%, respectively. In terms of relative contribution to the overall CS rate, Group 5 was the largest one, accounting for 36.0%. This was followed by Group 2, which accounted for 20.3%. Next was Group 4 (multiparous women with single cephalic pregnancy at term without previous CS who either had an induction of labor or CS before the onset of labor) (9.0%) and Group 1 (7.6%). These four groups accounted for over 75% of all CS in public healthcare. As for the size of Robson subgroups in public healthcare, of the 1271 women in Group 2, 289 (22.7%) had pre-labor CS (2b) and the remaining 982 (77.3%) were induced (2a). The 826 women in Group 4 subdivided into 688 (83.3%) who had induction (4a) and 138 (16.7%) who had pre-labor cesarean Sects. (4b). Group 10 accounted for 13.1% of all deliveries and 13.5% of C-sections (Table 3).

Robson Groups 6 to 9, accounted for 8.3% of women in private care and 4.4% in public healthcare. Their relative contributions to the overall CS rate were 10.1 and 9.4%, respectively (Table 3). These groups were not included in the following analysis due to non-cephalic presentation or multiple fetus pregnancy, and consequently, with a high (or full) probability of CS indication.

**Fig. 2:**
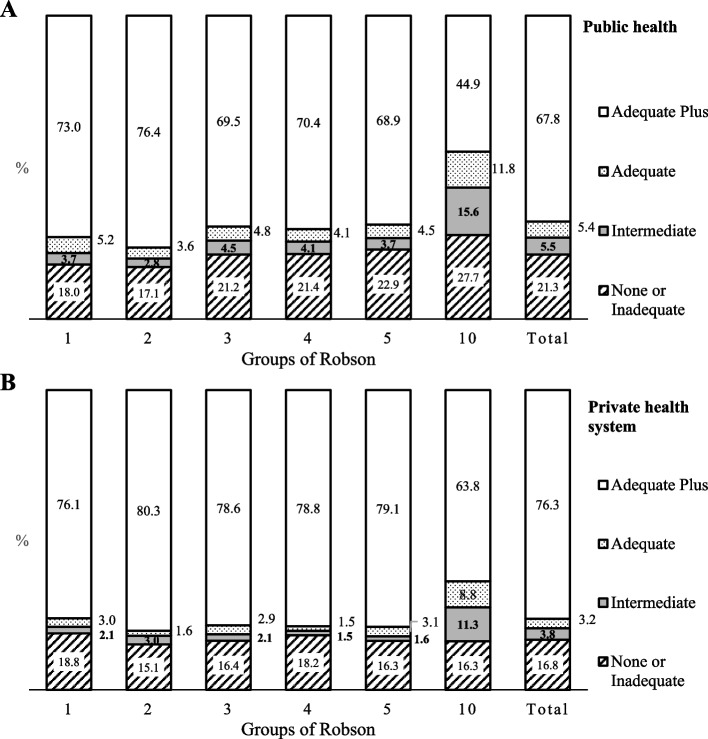
Access to prenatal care according to groups of Robson for public (**A**) and private (**B**) health systems

Figure 2 shows the groups of Robson according to APNCU index in public and private health systems. In general, 67.8 and 76.3% of women attended by public and private health system, respectively, received antenatal care classified as adequate plus. This percentage is much lower for those in group 10 (< 37 gestational weeks), with 44.9 and 63.8% for the public and private health systems, respectively. Notwithstanding, 27.7% of women attended in the public health system received no or inadequate care.

Table 4 shows data for the estimated CS rates of selected Robson Groups (1, 2, 3, 4, 5, and 10) by level of access to prenatal care (APNCU), adjusted for maternal age, schooling, marital status, and ethnicity/skin color and are described as follows: Group 1 within public healthcare had 24.7% in inadequate, 23.4% in intermediate, 26.4% in adequate, and 23.6% in adequate plus; adjusted CS rates in private healthcare were 61.6, 60.1, 63.8, and 60.3% for each APNCU category, respectively. Group 2 within public healthcare had adjusted CS rates between 49.7 and 53.7%. Private care adjusted CS rates for this subgroup ranged from 82.9 to 85.1%. Group 3 presents adjusted CS rates from 7.1% (intermediate and adequate plus) to 8.2% (adequate) in public healthcare, and from 27.2% (intermediate) to 30.5% (adequate) for private healthcare. Adjusted CS rates within public healthcare for Group 4 ranged from 27.9 to 31.2%; and in privately insured parturients, from 65.5 to 69.0%. Adjusted CS rates for Group 5 within public healthcare were 61.1% (intermediate) to 64.8% (adequate), and for private, 88.5% (intermediate) to 90.0% (adequate). And finally, Group 10 (all women with single cephalic pregnancy before term, including those with previous CS) for public healthcare adjusted CS rates spanned from 38.9% (intermediate) to 42.7% (adequate), and from 75.8% (intermediate) to 78.6% (adequate) within private care (Table 4).

**Table 4:** CS rate according to adequacy of prenatal care and groups of Robson and WHO expected values for each group [15], by healthcare system

		**Adjusted CS rate**^**a**^** (CI 95%)**		**WHO expected values**
**Robson Group**	**Adequacy of access to prenatal care**	**Health care system**		
		**Public**	**Private**	
	No prenatal care/Inadequate	24.7 (20.8—29.0)	61.6 (55.4—67.5)	
1	Intermediate	23.4 (18.5—29.2)	60.1 (52.0—67.6)	< 10%
	Adequate	26.4 (22.8—30.4)	63.8 (58.2—69.1)	
	Adequate plus	23.6 (18.7—29.4)	60.3 (52.3—67.8)	
	No prenatal care/Inadequate	51.4 (46.3—56.4)	83.8 (80.1—87.0)	
2	Intermediate	49.7 (42.7—56.7)	82.9 (77.9—87.0)	20 – 35%
	Adequate	53.7 (49.4—58.0)	85.1 (81.9—87.8)	
	Adequate plus	50.0 (43.0—57.0)	83.1 (78.0—87.1)	
	No prenatal care/Inadequate	7.5 (6.0—9.3)	28.5 (23.3—34.4)	
3	Intermediate	7.1 (5.3—9.4)	27.2 (21.1—34.4)	< 3%
	Adequate	8.2 (6.7—10.0)	30.5 (25.4—36.1)	
	Adequate plus	7.1 (5.4—9.5)	27.4 (21.2—34.6)	
	No prenatal care/Inadequate	29.2 (25.1—33.7)	67.0 (61.0—72.4)	
4	Intermediate	27.9 (22.5—34.0)	65.5 (57.8—72.5)	< 15%
	Adequate	31.2 (27.4—35.3)	69.0 (63.6—73.9)	
	Adequate plus	28.1 (22.7—34.2)	65.7 (58.0—72.7)	
	No prenatal care/Inadequate	62.6 (58.3—66.8)	89.2 (86.6—91.3)	
5	Intermediate	61.1 (54.5—67.3)	88.5 (84.9—91.4)	50 – 60%
	Adequate	64.8 (61.1—68.3)	90.0 (87.8—91.9)	
	Adequate plus	61.3 (54.8—67.5)	88.6 (85.0—91.4)	
	No prenatal care/Inadequate	40.5 (35.7—45.4)	76.9 (72.1—81.1)	
10	Intermediate	38.9 (32.9—45.2)	75.8 (69.7—80.9)	~ around 30%
	Adequate	42.7 (38.2—47.3)	78.6 (74.2—82.4)	
	Adequate plus	39.1 (32.9—45.7)	75.9 (69.7—81.2)	

*CS* Cesarean section, *CI* Confidence Interval, *WHO* World Health Organization

^a^Estimates calculated through adjusted logistic model, and the following variables were included: maternal age, schooling, marital status, and ethnicity/skin color. Adequacy of prenatal care were not statistically significant (*p* = 0.2508)

## Discussion

Our data did not show any statistically significant association between the adequacy of prenatal care – according to the APNCU index – and the rate of CS for any of the Robson Groups selected (1, 2, 3, 4, 5, and 10) in either public or private healthcare. Distinctively, while the CS rates vary little across APNCU categories, they are markedly different across some Robson Groups and between the types of health system. This suggests that the level of access to prenatal care as measured by the APNCU index is not a relevant factor behind the CS rates observed in our data.

The APNCU index evaluates the adequacy of prenatal care through the number of prenatal visits and gestational age at the first prenatal visit. For assessing a potential relationship between prenatal care and CS rates, perhaps this index is not appropriate to capture potentially relevant information. For instance, the APNCU category does not indicate if and how there were any discussions on methods of delivery during prenatal visits. Moreover, visits are often brief, with insufficient time for elaborating on such topic, especially in the public health system [20].

A recent systematic review evaluated measurement properties of 12 prenatal care indices [21]. According to this review, both the APNCU index and the Kessner index are supported by moderate evidence regarding their reliability, predictive, and concurrent validity. The indices were the two most utilized among the studies reviewed and presented the strongest evidence regarding their measurement properties. Nevertheless, Rowe et al. reported that there is insufficient research to inform the choice of a single best index [21].

A Brazilian study investigated associations between CS rates and different variables in the state of Rio de Janeiro from 2015–2016. Their results differ from ours in that they reported an association between CS rates and the level of prenatal care based on the APNCU index. As the category of prenatal care improved, the CS rate increased [22]. Besides from recruiting from different population samples and environments, there are also a few methodological differences between the two studies. Crucially, we explored the association between CS rates and APNCU categories by splitting Robson groups and maintaining only the ones deemed relevant to this analysis, calculated adjusted CS, and analyzed data according to health care system. Another Brazilian study also investigated the role of prenatal care as a factor to CS deliveries [20]. Because they did not use the Robson classification nor the APNCU index, comparisons between their work and ours is less appropriate. Nevertheless, it is worth noting that Fabbro et al. reported that six or more prenatal visits increased the probability of CS rates by 47%. In analyzing all such results, we consider that the number of prenatal visits and the first gestational age in which they occur might not contribute to decrease the number of CS deliveries. Thus, the APNCU index, on its own, does not seem to be a parameter worthy of targeting as part of a strategy to increase vaginal deliveries.

Recent CS rates reported in Brazil – from studies encompassing cities, states, and the whole country – vary between 43.5 and 60.3% [2, 5, 10, 20, 22–24]. Our study exhibits overall CS rates (48.8%) significantly higher than the global averages but within this range. The latest available data (2010–2018) from 154 countries covering 94.5% of world live births shows that 21.1% of women gave birth by CS worldwide, rates ranging from 5% in sub-Saharan Africa to 42.8% in Latin America and the Caribbean [14].

Analysis of the Robson Classification groups revealed an increased cesarean section rate in Group 2 (nulliparous women with induction of labor) and Group 5 (multiparous women with a previous cesarean section). Therefore, it is important to prepare the pregnant woman for induction of labor to reduce the possibility of cesarean section, thus avoiding its use in this group. As a consequence of avoiding the first cesarean section, a reduction of multiparous women with previous cesarean sections would be encountered, hence creating long-term benefits.

The examination of our Robson Report Table also reveals two general points. First, the rate of CS is much higher in private healthcare. Secondly, CS rates within public healthcare, despite being lower than in private care, were also elevated across all Robson Groups in comparison with global rates.

Regarding the first point, the very high CS rates in private care agreed with previous reports of Brazilian healthcare data [1, 5, 23]. For Robson Groups 1 and 2 (which encompass most nulliparas) CS rates within private care are more than two times higher than the ones observed in public healthcare. The relative difference in CS rates between private and public care were even higher within Groups 3 and 4 (most multipara with no previous CS) and reached almost threefold. A significant portion of such private–public differences stems from scheduled CS deliveries, that is, in advance of labor. In fact, the ratio of women in Group 2b as a proportion of Groups 1 and 2 was 48.6% in private care versus 12.9% within public healthcare. For Group 4b as a proportion of Groups 3 and 4, the ratio was 31.9% among privately insured parturients, while only 6.4% in public healthcare. Conversely, the proportion of induced vaginal deliveries (Groups 2a and 4a) in public healthcare is much higher than in private care, suggesting that inductions are part of typical conduct at the hospital. In fact, there is a salient difference between deliveries in private and public healthcare in our hospital that may help explain the higher prevalence of scheduled CS in private care. Privately insured parturients usually have their obstetric team of choice – typically the same professionals that followed them throughout prenatal care – and are, to a certain extent, subject to their schedule. In public healthcare parturients do not usually select the professionals involved. Instead, their deliveries are conducted by the on-call team.

The second general point worth highlighting about our findings is the fact that, even within public healthcare alone, CS rates for all groups are higher than global rates and WHO expected values. The rates for most Robson groups (1, 2, 3, 4) even in public healthcare were approximately two times greater than their respective WHO expected values. CS rates for Groups 5 and 10 were also above expected values but with minor differences (50–60% vs ~ 62%, and ~ 30% vs ~ 40%, respectively). There are probably several intertwined factors contributing to this scenario. Obstetric teams may be privileging unnecessary CS deliveries to save their time. Perhaps induction of labor is not being adequately offered to all pregnant women following Robson criteria. Very importantly, there are the choices that the parturients themselves are making. A recent study conducted in Brazil outlines additional elements that contribute to high rates of cesarean sections in the country. These include the absence of a collaborative approach among healthcare professionals in childbirth care, a limited availability of pharmacological pain relief (especially evident in public healthcare), unclear guidelines regarding the necessity of early delivery in cases of suspected fetal health issues, and insufficient financial support for obstetric care [25].

A national survey with 24,000 Brazilian pregnant women evaluated how preferences changed during pregnancy. It showed that while only 27.6% women in private care stated cesarean as their initial preference (at the beginning of pregnancy), 87.5% ended up delivering via CS. Fear of labor pain was the most cited reason for preferring a CS delivery, especially among nulliparous women [26]. Other studies have further explored this topic with similar findings [27–29].

Policies regarding CS deliveries in Brazil have been moving in the direction of giving women more power to decide their mode of delivery [30]. In conjunction with pro-choice rules, campaigns on the risks and benefits of each mode of delivery and additional counseling have been implemented across both public and private care in recent years. This seems fundamental in aiding women make well-informed decisions about their deliveries – such as *Rede Cegonha* (“Stork Network”) and *Parto Adequado* (“Adequate Delivery”). However, it is not yet possible to prove that such initiatives will, in fact, be able to modify CS rates from here on. A study by Cochrane reviewed 29 studies to evaluate the effectiveness of non-clinical interventions intended to reduce unnecessary CS. The evidence so far, although limited, indicates that prenatal-based programs have made little or no difference on CS rates [31]. There is therefore opportunity for future work to revisit this situation and investigate if these initiatives may affect CS rates.

We should mention that the cross-sectional design is a limitation of our study, which makes it impossible to verify causal associations. As additional limitations we can cite firstly that all deliveries considered here took place in the same hospital, PUC-Campinas. Although data are from a single hospital, all deliveries over a four-year period that contained the information of interest (98.5%) were analyzed, allowing characterization of a census for a tertiary hospital, a reference for high-risk pregnancy. Secondly, the use of data recorded during routine hospital work, although not specifically collected for the study, enabled data verification and minimal use of resources. Thirdly, prenatal visits took place in multiple clinics and healthcare facilities across the Campinas metropolitan area, therefore, the analyses considering adequacy of prenatal (APNCU levels) have limited comparability across public and private healthcare. Finally, no information with regards to maternal or fetal risk was considered (e.g., hypertensive disorders, eclampsia, preexisting diabetes, gestational diabetes, severe chronic diseases, infection at hospital admission for birth, placental abruption, placenta previa, intrauterine growth restriction and major newborn malformation). However, Robson classification utilizes epidemiological criteria to structure the ten groups, that take these prevalences into account.

## Conclusion

Our study investigated if there was association between CS rates and the adequacy level of prenatal care (via the APNCU index) and no statistically significant association was found. This lack of association clarifies the importance of adequate and qualified perinatal care, not just adequate access to prenatal care as measured by the APNCU index.

Another finding in our study was the significant differences in private versus public CS rates, considering the Robson groups, although for both healthcare systems the highest CS rate was for group 5 (multiparous women with a previous cesarean section), pointing efforts to avoid the first CS. Therefore, reducing unnecessary CS deliveries remains an elusive challenge.

## Data Availability

Data from SINASC-DATASUS are publicly available at: https://datasus.saude.gov.br/transferencia-de-arquivos/#. The data from Digitalized hospital records are not publicly available due to restrictions as they contain information that could compromise the privacy of research participants.

## Abbreviations

APNCU: Adequacy of the prenatal care utilization
CS: Cesarean sections
SUS: Sistema Único de Saúde – Brazilian National Public Health System
PUC: Pontifical Catholic University
WHO: World Health Organization
